# Blood Transfusion, Serum Ferritin, and Iron in Hemodialysis Patients in Africa

**DOI:** 10.1155/2015/720389

**Published:** 2015-01-11

**Authors:** Leonard Kouegnigan Rerambiah, Laurence Essola Rerambiah, Armel Mbourou Etomba, Rose Marlène Mouguiama, Phanie Brunelle Issanga, Axel Sydney Biyoghe, Batchelili Batchilili, Sylvestre Akone Assembe, Joel Fleury Djoba Siawaya

**Affiliations:** ^1^Centre National de Transfusion Sanguine (CNTS), Libreville, Gabon; ^2^Service d'Anesthésie-Réanimation du Centre Hospitalier Universitaire de Libreville, Libreville, Gabon; ^3^Centre National d'Hémodialyse de Libreville, Libreville, Gabon; ^4^Centre Hospitalier Universitaire d'Angondjè, Libreville, Gabon; ^5^Unité de Recherche et de Diagnostic Spécialisé/Laboratoire National de Santé Publique (URDS/LNSP), Libreville, Gabon

## Abstract

*Background and Objectives*. There is no data analyzing the outcome of blood transfusions and oral iron therapy in patients with kidneys failure in sub-Saharan Africa. The present study aimed to fill that gap and assess the value of ferritin in the diagnosis of iron overload and deficiency. *Design*. From January to February 2012, we prospectively studied 85 hemodialysis patients (78% of males and 22% of females aged 20 to 79 years) attending the Gabonese National Hemodialysis Centre. *Results*. Correlation studies showed (a) a strong positive linear relationship between the number of blood transfusions and high serum ferritin in hemodialysis patient (Spearman *r* : 0.74; *P* value: 0.0001); (b) a weak association between the number of blood transfusions and serum iron concentrations (Spearman *r* : 0.32; *P* value: 0.04); (c) a weak association between serum ferritin and serum iron (Spearman *r* : 0.32; *P* value: 0.003). Also, the strength of agreement beyond chance between the levels of ferritin and iron in the serum was poor (*κ* = 0.14). The prevalence of iron overload was 10.6%, whereas the prevalence of iron deficiency was 2.3%, comparing (1) patients with a maximum of one transfusion not on iron therapy; (2) patients with a maximum of one transfusion on iron therapy; (3) polytransfused patients not on iron therapy; and (4) polytransfused patients on oral iron therapy. The “Kruskal-Wallis test” showed that ferritin levels varied significantly between the groups (*P* value: 0.0001). *Conclusion*. Serum ferritin is not reliable as a marker of iron overload. For patients undergoing regular transfusion we recommend routine serum ferritin measurement and yearly measurement of LIC.

## 1. Introduction

Renal anemia due to iron-restricted erythropoiesis is common condition associated with chronic renal failure [1, 2]. Kidneys secrete erythropoietin, a protein involved in erythropoiesis. When kidneys are damaged, the secretion of erythropoietin decreases, resulting in renal anemia [3]. Because iron is also required for erythropoiesis, low iron may also cause anemia [3, 4]. Blood transfusion, erythropoietin (EPO), and iron therapy remain the principal means to treat renal anemia in most settings [3–6]. However, renal anemia correction in chronic renal failure patients not only carries a risk for iron overload [7, 8], but also increases the risk of adverse events such as hypertension, congestive heart failure, myocardial infarction, and vascular access thrombosis [3, 9].

Correction of renal anemia in the Gabonese setting continues for the big part to be done by iterative blood transfusions and oral iron therapy. Although transfusions are considerably safer nowadays [10], transfusion-related risks persist [11–13]. These risks include transmission of infectious agents [11, 14, 15], the development of alloimmunization [16, 17], and iron overload [18]. Additionally, significant costs are associated with blood transfusions and EPO therapy to which few patients have access.

Currently there is no data in sub-Saharan Africa analyzing the outcome of blood transfusions and oral iron therapy in patients with kidneys failure. The present study aimed to evaluate iron status in patients with renal failure undergoing hemodialysis and assess the value of ferritin in the diagnosis of iron overload and iron deficiency in an African setting.

## 2. Material and Methods

From January to February 2012, in a prospective cross-sectional study, we studied 85 hemodialysis patients (78% males and 22% females aged 20 to 79 years) attending the Gabonese National Hemodialysis Centre. Patients were divided into four (4) groups. Group one (1) consists of patients with zero (0) or one (1) transfusion under no iron therapy; group two (2) consists of patients with zero (0) or one (1) on iron therapy; group three (3) consists of polytransfused patients under no iron therapy; group four (4) consists of polytransfused patients on oral iron therapy.

### 2.1. Sample Handling

Blood samples were taken before the hemodialysis session. 5 mL of venous blood was collected into plain tubes. The collected blood samples were then centrifuged (5000 rev/min for 10 minutes) and sera stored at −80°C pending analysis.

### 2.2. Enzyme-Linked Fluorescence Assay

Commercially available ELFA kits were used to measure iron and ferritin in the serum (Biomérieux, France). Samples were assayed according to the manufacturer's protocol. Samples were read using a mini-VIDAS reader set to 450 nm. The mini-VIDAS system software generated concentrations of the respective analytes.

### 2.3. Data Analysis

Table 1 shows ferritin and iron concentration ranges in the serum and the corresponding interpretations. The strength of agreement between serum concentrations of ferritin and iron was assessed based on the Kappa coefficient (*κ*) that was calculated using the formula *κ* = (*P*_*o*_ − *P*_*e*_)/1 − *P*_*e*_, where *P*_*o*_ is the relative observed agreement among raters and *P*_*e*_ is the hypothetical probability of chance agreement. All other statistical analyses were performed using Prism 6 software from GraphPad Software (San Diego, California, USA). The differences between the groups were analyzed using the Kruskal-Wallis multiple comparison test and Dunn's post test. A *P* value below 5% was considered significant.

### 2.4. Ethics

The National Laboratory of Public Health board and the National Blood Transfusion Centre board approved the study. All patients consented to participate in the study.

## 3. Results

### 3.1. Correlation between Transfusion, Ferritin, and Iron Levels

**Figure 1:**
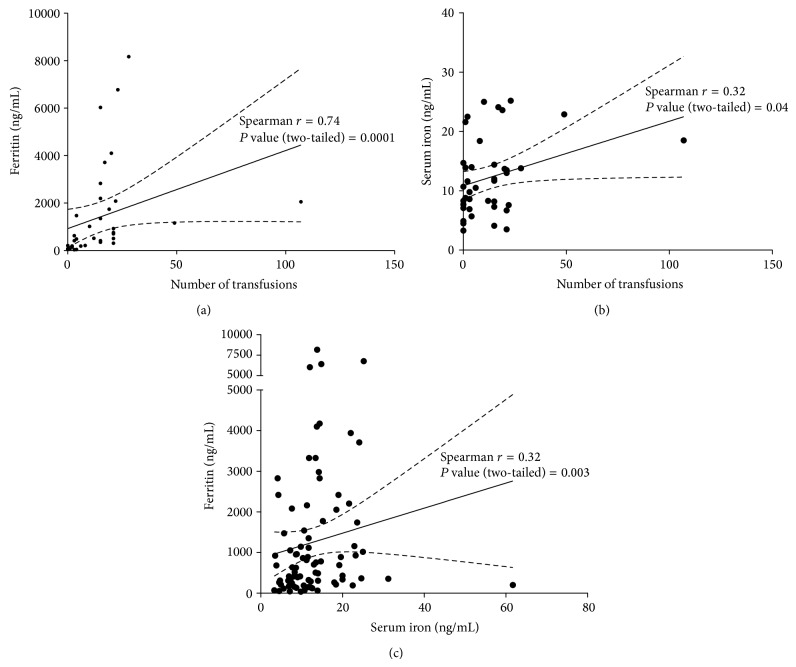
Correlation between (a) serum ferritin and the number of blood transfusions: the graph shows strong positive linear relationship between the number of blood transfusions and high serum ferritin in hemodialysis patient (Spearman *r* : 0.74; *P* value: 0.0001); (b) serum iron and the number of blood transfusions: the graph shows a weak association between the number of blood transfusions and serum iron concentrations (Spearman *r* : 0.32; *P* value: 0.04); (c) serum ferritin and serum iron: the graph shows a weak association between serum ferritin and serum iron (Spearman *r* : 0.32; *P* value: 0.003).

Data showed a positive and significant correlation between the number of transfusions and ferritin levels (Spearman *r* : 0.74; *P* value: 0.0001) (Figure 1(a)). Although weak, the correlation between the number of transfusions and serum iron levels was positive and significant (Spearman *r* : 0.32; *P* value: 0.04) (Figure 1(b)). Further correlation analysis showed a significant association between serum ferritin and serum iron concentrations (Spearman *r* : 0.32; *P* value: 0.003) (Figure 1(c)). However, the observed Spearman *r* coefficient suggested that although significant, the correlation between serum ferritin and serum iron concentrations is weak.

### 3.2. Strength of Agreement between Ferritin and Iron Levels in Establishing Iron Overload

Table 2 shows patients distribution according to their ferritin and iron levels in our setting. The prevalence of iron overload based on serum ferritin was 42.3%. When assessed based on serum iron level iron overload prevalence was 21.2%. The prevalence of iron overload based on both ferritin and iron concentration in the serum was 10.6%. The prevalence of iron deficiency based on serum ferritin was 17.4%, whereas the prevalence of iron deficiency based on serum iron concentration was 10.6%. 2.3% was the prevalence of iron deficiency when both ferritin and iron concentration were crossed.

Applying Cohen's Kappa (*κ*) formula we got a *κ* coefficient of 0.14. Therefore strength of agreement beyond chance between the levels of ferritin and iron in the serum was weak or poor.

### 3.3. Transfusion and Serum Ferritin and Iron

**Figure 2:**
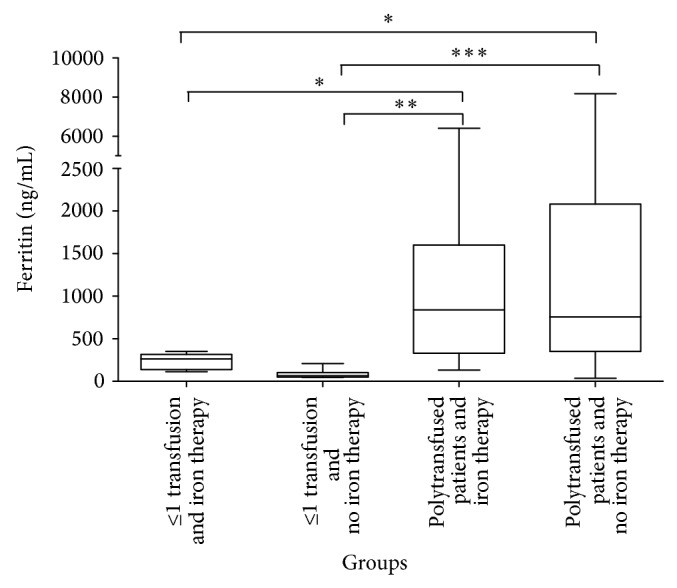
Serum ferritin levels in (1) patients with zero or one transfusion under no iron therapy; (2) patients with zero or one under iron therapy; (3) polytransfused patients under no iron therapy; (4) polytransfused patients under oral iron therapy. The star (∗) marks significant differences between groups. ^***^ *P* value of 0.0001; ^**^ *P* value of 0.001; ^*^ *P* value < 0.05.

Comparing patients with zero (0) or one (1) transfusion (on iron therapy and not on iron therapy) to polytransfused patients (on iron therapy and not on iron therapy), the “Kruskal-Wallis test” showed that ferritin levels varied significantly between the groups (*P* value: 0.0001). “Dunn's Multiple Comparison Test” showed that (1) in patients not on iron therapy ferritin level was significantly higher in polytransfused patients than patients with zero (0) or one (1) transfusion (*P* value: 0.0001), (2) in patients on iron therapy ferritin level was significantly higher in polytransfused patients than patients who had zero (0) or one (1) transfusion (*P* value < 0.05), (3) polytransfused patients on iron therapy had significantly higher levels of ferritin compared to patients who had zero (0) or one (1) transfusion who were not on iron therapy (*P* value: 0.001), and (4) polytransfused patients who were not on iron therapy had significantly higher levels of ferritin compared to patients who had zero (0) or one (1) transfusion and who were on iron therapy (*P* value < 0.05) (Figure 2). Comparing the same groups of patients for their serum iron concentrations, the “Kruskal-Wallis test” showed no significant differences between the groups.

## 4. Discussion

In the developing world the serum level of ferritin in patients is still used as the principal marker for diagnosis of iron overload or deficiency in hemodialysis patients. Monitoring iron status using serum ferritin may be subtle for hemodialysis services workers, as there are confounding factors such as acute, chronic inflammation and malnutrition that could lead to differential when interpreting serum ferritin values [19]. Here we showed a strong positive linear relationship between the number of blood transfusions and high levels of serum ferritin. We also showed a weak association between the number of blood transfusions and the concentrations of iron in the serum. The strength of agreement between ferritin and iron levels in the serum was poor. Data suggests that multiple transfusions increase substantially serum ferritin and to a lesser extent serum iron.

We found that in hemodialysis patients serum ferritin greatly overestimates iron burden. Serum ferritin overestimates also iron deficiency, but to a lesser extent. Indeed, in our setting, the prevalence of iron overload based on serum ferritin was 42.3 whereas the prevalence of iron overload based on serum iron levels was 21.2%. The prevalence of iron deficiency based on serum ferritin and serum iron was, respectively, 17.4% and 10.6%. Data also showed that 65% of patient who had moderately high serum ferritin levels and 67% of patients who had very high serum ferritin levels had their serum iron level within the normal range. Therefore, our results denote and confirmed that high serum ferritin is not a reliable marker of iron overload [20, 21].

Because the use of serum ferritin as marker for iron overload or deficiency could lead to (1) withholding iron therapy in patients that need it and (2) giving iron treatment to patients who do not require it, accurate assessment of the body iron load is essential to prevent iron toxicity and to manage iron chelation therapy. Although we did not assess liver iron concentration (LIC) by magnetic resonance imaging (MRI), based on the published report [3, 22–25], we could recommend it for the management of hemodialysis patients in developing countries. However, MRI-based methods are still relatively expensive in that part of the world. Therefore the accurate diagnosis and management of iron overload in developing countries remain challenging. In these countries, for patients undergoing regular transfusion therapy we would suggest to associate with the routine serum ferritin measurement, a yearly measurement of LIC as proposed by Hoffbrand and colleagues [22].

## Figures and Tables

**Table 1:** Ferritin and iron ranges in the serum and interpretations.

	Iron deficiency	Normal	Indeterminate (moderately high)	Iron overload
Serum ferritin	<100 ng/mL		200–800 ng/mL	>800 ng/mL
Serum iron	<4 ng/mL	5–15 ng/mL		>15 ng/mL

**Table 2:** Patients' distribution according to their ferritin and iron levels in the serum.

	Serum ferritin			
	Iron deficiency (<100 ng/mL)	Indeterminate (200–800 ng/mL)	Iron overload (>800 ng/mL)	Total
Serum iron				
Iron deficiency (<4 ng/mL)	2	4	3	9
Normal (5–15 ng/mL)	12	22	24	58
Iron overload (>15 ng/mL)	1	8	9	18
Total	**15**	**34**	**36**	**85**
